# Supplementary material for: Chirality detection of enantiomers using twisted optical metamaterials
Source: Nat Commun. 2017 Jan 25;8:14180. doi: 10.1038/ncomms14180 (PMC5288493; doi:10.1038/ncomms14180)
Supplement: Supplementary Information — Supplementary Figures, Supplementary Notes and Supplementary References. [file ncomms14180-s1.pdf]

Supplementary Note 1. Frequency shift from circular dichroism measurements

We model the chiral molecules adsorbed on the surface of the metamaterial as a thin homogeneous chiral film with thickness *w*. The loaded metamaterial is excited separately with a right-handed (R) and a left-handed (L) circularly polarized plane wave. The wavenumber of the incident field is *k* in the molecular layer. The output from the metamaterial is composed of both left- and right-handed plane waves, which is considered as the input to the chiral film. We define the circular dichroism of the wave exiting the metamaterial and before entering the chiral film as CD_i:

$$CD_\mathrm{i} = \tan^{-1}\frac{I_\mathrm{R}-I_\mathrm{L}}{I_\mathrm{R}+I_\mathrm{L}} = \tan^{-1}\frac{|T_\mathrm{LR}|^2+|T_\mathrm{RR}|^2-|T_\mathrm{RL}|^2-|T_\mathrm{LL}|^2}{|T_\mathrm{LR}|^2+|T_\mathrm{RR}|^2+|T_\mathrm{RL}|^2+|T_\mathrm{LL}|^2}\,, \tag{1}$$

where I_R and I_L denote the total transmitted power of the plane waves with right- and left-handed excitations. The circular dichroism after the chiral film can be readily calculated by applying the electromagnetic boundary conditions for a chiral film of width *w*. Expanding the output circular dichroism in terms of *kw* and keeping only the first two leading terms provided $kw \ll 1$, we obtain

$$CD_\mathrm{o} = CD_\mathrm{i} + 4kw\,\mathrm{Im}[\kappa_\mathrm{m}]\frac{|T_\mathrm{LR}T_\mathrm{RL}|^2-|T_\mathrm{LL}T_\mathrm{RR}|^2}{\left(|T_\mathrm{LR}|^2+|T_\mathrm{RR}|^2\right)^2+\left(|T_\mathrm{LL}|^2+|T_\mathrm{RL}|^2\right)^2}\,, \tag{2}$$

where κ_m is the chirality of the chiral molecule film. By expanding CD_o near the resonance ($k_0 = 2\pi/\lambda_0$, with λ_0 being the resonance free-space wavelength of CD_i), we obtain

$$CD_{\mathrm{o}} \approx CD_{\mathrm{i}}^{(0)} + \frac{CD_{\mathrm{i}}^{(2)}}{2}\left(k-k_0\right)^2 + 4kw\,\mathrm{Im}\left[\kappa_{\mathrm{m}}\right]\frac{\left|T_{\mathrm{LR}}T_{\mathrm{RL}}\right|^2 - \left|T_{\mathrm{LL}}T_{\mathrm{RR}}\right|^2}{\left(\left|T_{\mathrm{LR}}\right|^2 + \left|T_{\mathrm{RR}}\right|^2\right)^2 + \left(\left|T_{\mathrm{LL}}\right|^2 + \left|T_{\mathrm{RL}}\right|^2\right)^2}, \quad (3)$$

where $CD_{\mathrm{i}}^{(2)}$ represents the second derivative of CD_{i} with respect to k_0. From equation (3), one can find the shift in resonance frequency of CD_{o} with respect to the resonance frequency of the CD_{i}. The frequency shift is

$$\Delta\omega = c\frac{4\,\mathrm{Im}\left[\kappa_{\mathrm{m}}\right]w}{CD_{\mathrm{i}}^{(2)}}\frac{\left|T_{\mathrm{RR}}\right|^2\left|T_{\mathrm{LL}}\right|^2 - \left|T_{\mathrm{LR}}\right|^2\left|T_{\mathrm{RL}}\right|^2}{\left(\left|T_{\mathrm{LR}}\right|^2 + \left|T_{\mathrm{RR}}\right|^2\right)^2 + \left(\left|T_{\mathrm{LL}}\right|^2 + \left|T_{\mathrm{RL}}\right|^2\right)^2}. \quad (4)$$

This equation shows that the sign of the frequency shift is reversed either due to a different sign of $CD_{\mathrm{i}}^{(2)}$ or due to a sign change in $\mathrm{Im}\left[\kappa_{\mathrm{m}}\right]$, which implies that the frequency shift will be opposite for the same chiral molecules on top of chiral metamaterials with opposite handedness, or opposite for S and R chiral molecules on top of the same metamaterial.

Supplementary Note 2. Circular dichroism and enhancement factors

The equations (2)-(4) are obtained assuming that the chirality coefficient of the molecular layer is known, and it neglects the near-field interaction between the metamaterial inclusions and the chiral molecules. Here, we incorporate the field and chiral enhancement factors in calculating the total output circular dichroism. Our analysis in the following is based on the assumption that the near-field interaction between molecules and electromagnetic fields can be modeled assuming plane wave propagation in the molecular layer, and adjusting the effective permittivity and chirality of this layer as a function of the enhanced local density of states near the metamaterial surface. This

adjustment is based on a comparison between near- and far-field effects of the twisted metamaterial. The imaginary parts of permittivity and chirality are respectively scaled according to the field and chiral enhancement factors in the near-field. This scaling can be originated from poynting's theorem and reciprocity theorem that the power loss density in a chiral medium is related to two parts. The first part arises from the loss embedded in the permittivity of the chiral molecules, which is proportional to the product of the imaginary part of permittivity and the intensity of the field ($\mathrm{Im}[\varepsilon]|\mathbf{E}|^2$). The second part is proportional to the product of the imaginary part of chirality of the molecules and the chirality of the field ($\mathrm{Im}[\kappa_{\mathrm{m}}]\mathrm{Im}[\mathbf{E}\cdot\mathbf{H}^*]$).[1]

The near-field enhancement factor $\mathscr{F}$ is defined as the near-field intensity within the vicinity of the metamaterial surface normalized to the far-field intensity,

$$\mathscr{F} = \frac{\left|\mathbf{E}_{\text{Near-Field}}\right|^2}{\left|\mathbf{E}_{\text{Far-Field}}\right|^2}. \tag{5}$$

The amount of loss due to the imaginary part of the permittivity for a molecular layer placed in the near-field of the twisted metamaterial can be expressed as $\mathscr{F}\,\mathrm{Im}\left[\varepsilon\right]\left|\mathbf{E}\right|^2$, where the electric field is a far-field quantity. It is seen that we can always converge to the same amount of loss by either keeping the field enhancement factor inside $\left|\mathbf{E}\right|^2$, or embedding this enhancement factor inside the imaginary part of the permittivity while keeping $\left|\mathbf{E}\right|^2$ unchanged as a far-field plane wave, introducing an effective permittivity that accounts for the near-field enhancement. Similarly for the losses due to the imaginary part of chirality, one can define the chiral enhancement factor as $\mathscr{K}$. Here $\mathscr{K}$ is defined as the maximum chirality at the near-field of the metamaterial normalized to the maximum of the far-field chirality, where the field chirality is defined as $C = \mathrm{Im}[\mathbf{E}\cdot\mathbf{H}^*]$:[2]

$$\mathscr{K} = \frac{\mathrm{Im}[\mathbf{E}\cdot\mathbf{H}^*]_{\text{Near-Field}}}{\mathrm{Im}[\mathbf{E}\cdot\mathbf{H}^*]_{\text{Far-Field}}}. \tag{6}$$

Again, the loss can be expressed as the effect of the actual near-field chirality C interacting with $\mathrm{Im}[\kappa_{\mathrm{m}}]$, or equivalently it can be stated that the loss is due to the interaction between far-field plane waves with a material with effective chirality coefficient $\mathscr{K}\,\mathrm{Im}[\kappa_{\mathrm{m}}]$.

The coefficient κ_{m} is associated with the intrinsic CD response of the chiral molecules, but its effective value can be largely boosted by the near-field interaction with the plasmonic particles forming the metamaterial. The complex refractive index of the chiral molecules can be written as:

$$\begin{aligned} n_{\mathrm{LCP}} &= \sqrt{\varepsilon_{\mathrm{L}}} + \kappa_{\mathrm{mL}} = a + ib_{\mathrm{L}} - (c + id_{\mathrm{L}}) \\ n_{\mathrm{RCP}} &= \sqrt{\varepsilon_{\mathrm{R}}} + \kappa_{\mathrm{mR}} = a + ib_{\mathrm{R}} + (c + id_{\mathrm{R}}) \end{aligned}, \tag{7}$$

where the subscript denotes the polarization of the plane waves propagating in the chiral film. In the above equation, the parameters *a*, *b*, *c* and *d* are purely real numbers, associated with the real and imaginary parts of effective permittivity and chirality. As discussed above, here the near-field effects manifest themselves in the imaginary parts of effective permittivity and chirality of the film and therefore, the real parts of these two quantities are assumed to be equal for both right and left-handed impinging waves.

In conventional CD measurements, in which the chiral molecules interact with the impinging circularly polarized light without any contribution from the substrate, the refractive index of the chiral molecular film experienced by light is only different by the sign of κ_{m}; in other words, since there are no enhancement factors involved ($\mathscr{F} = 1, \mathscr{K} = 1$) we have $b_{\mathrm{L}} = b_{\mathrm{R}}$ and $d_{\mathrm{L}} = d_{\mathrm{R}}$ in equation (7). For CD measurements with chiral molecules adsorbed on *achiral* plasmonic structures, the

near-field enhancement factor $\mathscr{F}$ leads to $b_{\mathrm{L}} \neq b_{\mathrm{R}}$, but the lack of a chirality enhancement factor ($\mathscr{K}$ = 1) results in $d_{\mathrm{L}} = d_{\mathrm{R}}$. $\mathscr{F}$ is embedded in the imaginary terms of the relative permittivity, and thus can affect the absorption for circularly polarized light by the chiral molecules, such that $b_{\mathrm{L}} = b \cdot \mathscr{F}_{\mathrm{L}}$ and $b_{\mathrm{R}} = b \cdot \mathscr{F}_{\mathrm{R}}$. Notice that the field enhancement factor differs for different handedness of the circularly polarized light due to their different interactions with the chiral molecules. In addition to $\mathscr{F}$, plasmonic *chiral* metamaterials introduce an additional chiral enhancement factor $\mathscr{K}$,[3,4] resulting in $b_{\mathrm{L}} \neq b_{\mathrm{R}}$ and $d_{\mathrm{L}} \neq d_{\mathrm{R}}$, where $d_{\mathrm{R}} = d \cdot \mathscr{K}_{\mathrm{R}}$, $d_{\mathrm{L}} = d \cdot \mathscr{K}_{\mathrm{L}}$, respectively.

Since one particular polarization can be selectively enhanced to a larger degree in the near field, its effective losses inside the chiral film will also be larger compared to the other polarization. Therefore, CD_{o} can be revised by embedding these enhancement terms as

$$
\begin{aligned}
& CD_{\mathrm{o}} = CD_{\mathrm{i}} + \\
& -kw\,\mathrm{Re}\left[\sqrt{\varepsilon_{\mathrm{r}}}\right]\mathrm{Im}\left[\sqrt{\varepsilon_{\mathrm{r}}}\right](\Sigma_{\mathrm{R}} - \Sigma_{\mathrm{L}})\frac{\left(|T_{\mathrm{LR}}|^2 + |T_{\mathrm{RR}}|^2\right)\left(|T_{\mathrm{LL}}|^2 + |T_{\mathrm{RL}}|^2\right)}{\left(|T_{\mathrm{LR}}|^2 + |T_{\mathrm{RR}}|^2\right)^2 + \left(|T_{\mathrm{LL}}|^2 + |T_{\mathrm{RL}}|^2\right)^2} \\
& +kw\left(\mathrm{Im}\left[\sqrt{\varepsilon_{\mathrm{r}}}\right]\Delta_{\mathrm{R}} + 2\,\mathrm{Im}[\kappa_{\mathrm{m}}]\mathscr{K}_{\mathrm{R}}\right)\frac{\left(|T_{\mathrm{LR}}|^2 - |T_{\mathrm{RR}}|^2\right)\left(|T_{\mathrm{RL}}|^2 + |T_{\mathrm{LL}}|^2\right)}{\left(|T_{\mathrm{LR}}|^2 + |T_{\mathrm{RR}}|^2\right)^2 + \left(|T_{\mathrm{LL}}|^2 + |T_{\mathrm{RL}}|^2\right)^2}, \\
& +kw\left(\mathrm{Im}\left[\sqrt{\varepsilon_{\mathrm{r}}}\right]\Delta_{\mathrm{L}} + 2\,\mathrm{Im}[\kappa_{\mathrm{m}}]\mathscr{K}_{\mathrm{L}}\right)\frac{\left(|T_{\mathrm{LR}}|^2 + |T_{\mathrm{RR}}|^2\right)\left(|T_{\mathrm{RL}}|^2 - |T_{\mathrm{LL}}|^2\right)}{\left(|T_{\mathrm{LR}}|^2 + |T_{\mathrm{RR}}|^2\right)^2 + \left(|T_{\mathrm{LL}}|^2 + |T_{\mathrm{RL}}|^2\right)^2}
\end{aligned}
\tag{8}
$$

where

$$
\begin{aligned}
\Sigma_{\mathrm{R}} &= \mathscr{F}_{\mathrm{RR}} + \mathscr{F}_{\mathrm{LR}} \\
\Sigma_{\mathrm{L}} &= \mathscr{F}_{\mathrm{RL}} + \mathscr{F}_{\mathrm{LL}} \\
\Delta_{\mathrm{R}} &= \mathscr{F}_{\mathrm{RR}} - \mathscr{F}_{\mathrm{LR}} \\
\Delta_{\mathrm{L}} &= \mathscr{F}_{\mathrm{RL}} - \mathscr{F}_{\mathrm{LL}}
\end{aligned}
\quad ,
\tag{9}
$$

where $\mathscr{F}_{\mathrm{LR}}$ denotes the enhancement in the intensity of left-handed electric field near the surface of the metamaterial for a right-handed impinging wave. A similar convention is used for the other enhancement factors, as well as for the transmission coefficients. $\mathscr{K}_{\mathrm{R}}$ and $\mathscr{K}_{\mathrm{L}}$ are chiral enhancement factors for right and left-handed incident waves, respectively. To cancel the effective losses in CD_{o}, we employ both right handed (+) and left handed (-) metamaterials. By taking advantages from symmetries, such as $CD_{\mathrm{i}}^{+} = -CD_{\mathrm{i}}^{-}$, $T_{\mathrm{RR}}^{+} = T_{\mathrm{LL}}^{-}$, $T_{\mathrm{LR}}^{+} = T_{\mathrm{RL}}^{-}$, $T_{\mathrm{RL}}^{+} = T_{\mathrm{LR}}^{-}$, $T_{\mathrm{LL}}^{+} = T_{\mathrm{RR}}^{-}$, $\Sigma_{\mathrm{R}}^{+} = \Sigma_{\mathrm{L}}^{-}$, $\Sigma_{\mathrm{L}}^{+} = \Sigma_{\mathrm{R}}^{-}$, $\Delta_{\mathrm{R}}^{+} = -\Delta_{\mathrm{L}}^{-}$, $\Delta_{\mathrm{L}}^{+} = -\Delta_{\mathrm{R}}^{-}$, where the superscript represents the corresponding handedness of the metamaterial, one can obtain

$$\frac{CD_{\mathrm{o}}^{+} + CD_{\mathrm{o}}^{-}}{4kw\,\mathrm{Im}\left[\kappa_{\mathrm{m}}\right]} P = \mathscr{K}_{\mathrm{R}}^{+}\left(\left|T_{\mathrm{RL}}^{+}\right|^{2} + \left|T_{\mathrm{LL}}^{+}\right|^{2}\right)\left(\left|T_{\mathrm{LR}}^{+}\right|^{2} - \left|T_{\mathrm{RR}}^{+}\right|^{2}\right) + \mathscr{K}_{\mathrm{L}}^{+}\left(\left|T_{\mathrm{RL}}^{+}\right|^{2} - \left|T_{\mathrm{LL}}^{+}\right|^{2}\right)\left(\left|T_{\mathrm{LR}}^{+}\right|^{2} + \left|T_{\mathrm{RR}}^{+}\right|^{2}\right), \quad (10)$$

where

$$P = \left(\left|T_{\mathrm{LR}}^{+}\right|^{2} + \left|T_{\mathrm{RR}}^{+}\right|^{2}\right)^{2} + \left(\left|T_{\mathrm{LL}}^{+}\right|^{2} + \left|T_{\mathrm{RL}}^{+}\right|^{2}\right)^{2}, \quad (11)$$

which is equation (4) in the main text. Note that the effects from the field enhancement and losses arisen from the permittivity are cancelled out in this manipulation and the remaining terms are only dependent on the imaginary part of κ_{m} and the chiral enhancement factors.

It is worth noting that, due to fabrication imperfections, CD_{i}^{+} may not completely cancel out CD_{i}^{-} from the metamaterials substrates. In this scenario, equation (10) can be modified to

$$\frac{\left(CD_{\mathrm{o}}^{+} - CD_{\mathrm{i}}^{+}\right) + \left(CD_{\mathrm{o}}^{-} - CD_{\mathrm{i}}^{-}\right)}{4kw\,\mathrm{Im}\left[\kappa_{\mathrm{m}}\right]} P = \mathscr{K}_{\mathrm{R}}^{+}\left(\left|T_{\mathrm{RL}}^{+}\right|^{2} + \left|T_{\mathrm{LL}}^{+}\right|^{2}\right)\left(\left|T_{\mathrm{LR}}^{+}\right|^{2} - \left|T_{\mathrm{RR}}^{+}\right|^{2}\right) + \mathscr{K}_{\mathrm{L}}^{+}\left(\left|T_{\mathrm{RL}}^{+}\right|^{2} - \left|T_{\mathrm{LL}}^{+}\right|^{2}\right)\left(\left|T_{\mathrm{LR}}^{+}\right|^{2} + \left|T_{\mathrm{RR}}^{+}\right|^{2}\right)$$

. The subtraction of the CD_{i} removes system errors that stem from any differences in CD_{i} due to

fabrication, leaving the summation still directly related to the *signs* of the molecular chirality. Note that our fabrication indeed causes slight differences in the chirality enhancement factor $\mathscr{K}$ of the + and -60° metamaterials, which cannot be completely removed through this subtraction procedure. The above equation shows that the ∑CD is a function of the transmission coefficients as well as the chiral enhancement factor $\mathscr{K}$. In our case, the bare -60° metamaterial has a relatively stronger signal (as shown in Figure 2, where the magnitude of CD from the -60° metamaterial is larger than that from the +60° metamaterial). This indicates that $\mathscr{K}_L^- > \mathscr{K}_R^+$, resulting in a final ∑CD of the R-enantiomers (or all right-handed molecules) with a bigger magnitude compared to their left-handed enantiomeric pair at the same concentration. Please note that this systematic error is the same for a given metamaterial substrate, and it can be calibrated using a known molecule. One can therefore view Figure 3 as a calibration curve for our metamaterial substrates.

Supplementary Note 3. Fabrication of twisted metamaterials with different twist angles

We constructed a series of twisted metamaterials composed of two stacked metasurfaces separated by an 80nm electron-beam evaporated silicon dioxide layer, with densely packed nanorod inclusions. Five twisted metamaterials were fabricated with different twisting angles from the bottom layer metasurface to the top layer (see inset in Supplementary Figure 1), spanning from ±30° to ±90° at an interval of ±15°. The measured transmission spectra for positive angles are shown in Supplementary Figure 1**a**, with insets showing the top view scanning electron microscope (SEM) images of the corresponding metamaterials; these results are confirmed with full-wave numerical simulations in Supplementary Figure 1**b**. It is seen how the first four samples produce a different response for different circularly polarized inputs, reflected in the transmission

spectra and shown in Supplementary Figure 1**c** (experiment) and Supplementary Figure 1**d** (simulations). By changing the relative twist angle between the two layers, both the peak wavelength and maximum value of *CD* may be readily tuned and optimized (Supplementary Figure 1**d**). Supplementary Figure 1 confirms that the 60° twisted metamaterial provides the largest CD_i to boost chirality detection in molecules.

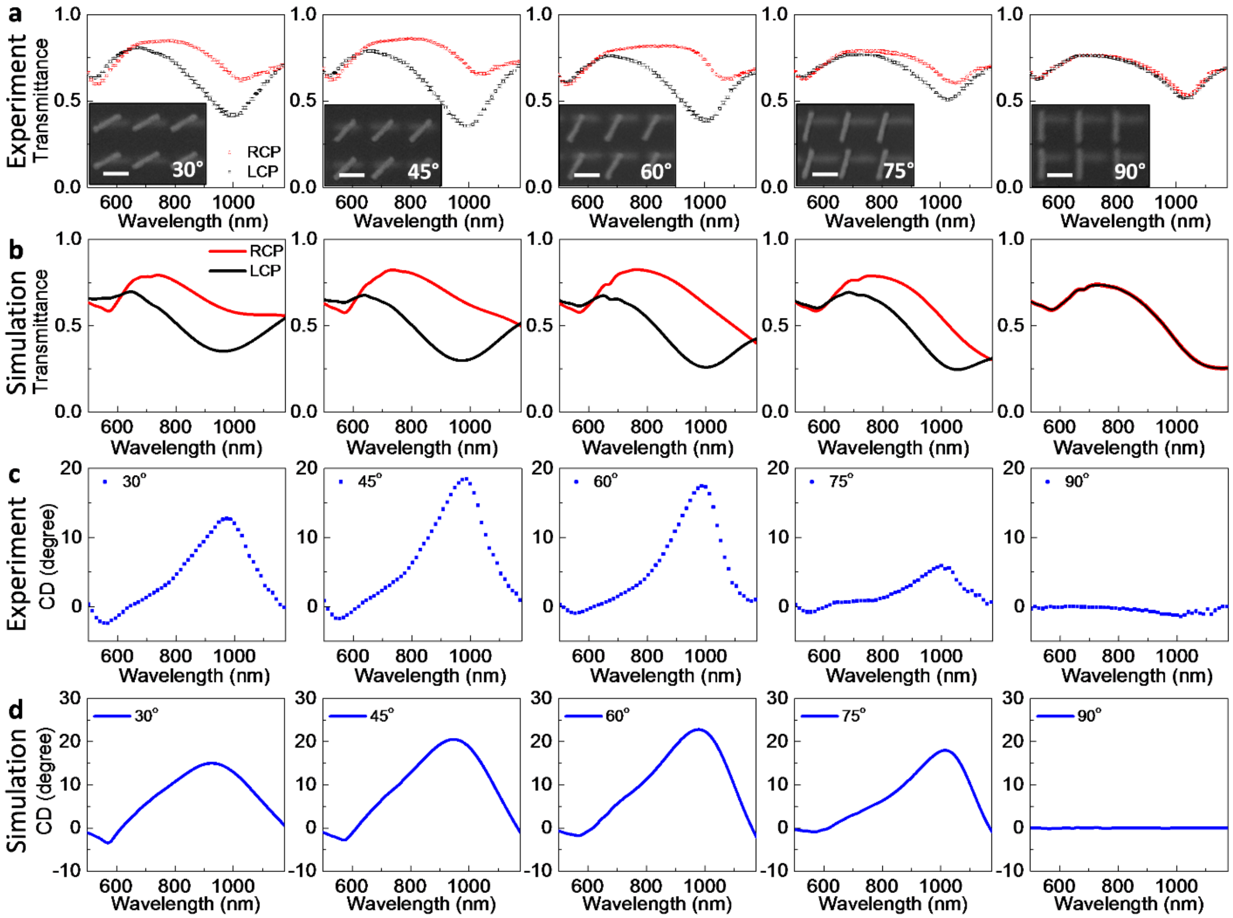

Supplementary Figure 1| Measurement and simulation of bare twisted metamaterials to create different chiral response. a, Experimental transmission measurements of twisted metamaterials with twist angles of 30°, 45°, 60°, 75° and 90° for RCP (red curves) and LCP (black curves) excitation (no analyzers are used). The twist angle is defined as the angle between the

nanorods unit cells between the two stacked layers, while keeping the lattice periodicity unchanged. Scanning electron microscope (SEM) images are shown in the insets. **b**, Corresponding full-wave numerical simulations of the transmission spectrum of these twisted metamaterials. **c**, Extracted circular dichroism from the measured transmission spectra. **d**, Corresponding numerical simulations of the circular dichroism of these twisted metamaterials. CD is defined as equation (1) in degrees.

Supplementary Note 4. Circular dichroism of tested molecules with conventional CD spectroscopy

We measured CD spectra of (S)-(+)-1,2-Propanediol and (R)-(−)-1,2-Propanediol on a commercial CD spectrometer (Jasco J-815), with a path length of 1 mm. The scan wavelength is limited from 195 nm to 260 nm due to the narrow bandwidth of the detector, as well as strong absorption below 195 nm that generates strong noises masking the CD signal. All the data were averaged over 3 measurements, as shown in Supplementary Figure 2. Conventional CD spectroscopy measurements of Concanavalin A and Irinotecan Hydrochloride can be found in the literature[5,6].

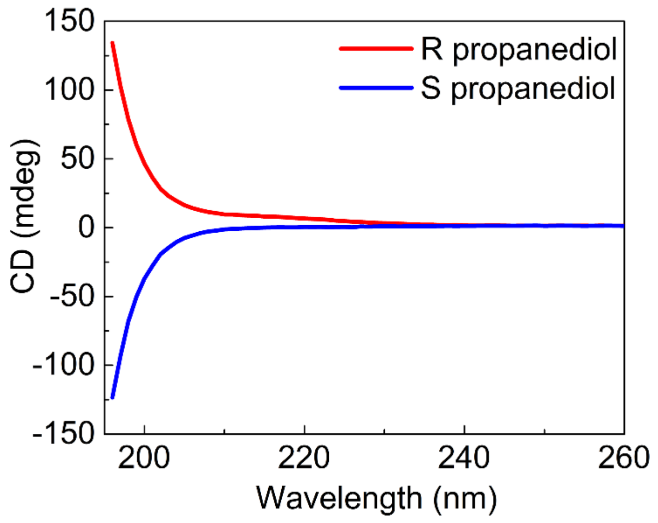

Supplementary Figure 2| CD spectrum of 1,2-Propanediol. CD spectrum in millidegrees of enantio-pure (S)-(+)-1,2-Propanediol (blue curve) and (R)-(-)-1,2-Propanediol (red curve) measured over wavelength range from 195 nm to 260 nm with a commercial CD spectrometer (Jasco J-815).

Supplementary Note 5. Near field profile and chiral enhancement as a function of distance from surface

In Supplementary Figure 3**a**, we show the optical chirality density at 2 nm above the top layer of the +60° twisted metamaterial, at 990 nm with right handed circularly polarized excitation, normalized to the far field. It is seen that the maximum of this optical chiral enhancement factor is around 40. In Supplementary Figure 3 panels **b** and **c**, we show how this chiral enhancement factor decays exponentially away from the top layer of the metamaterials. The integrated chiral enhancement factor (Supplementary Figure 3**c**) is the summation of all the enhancement factors at the same height.

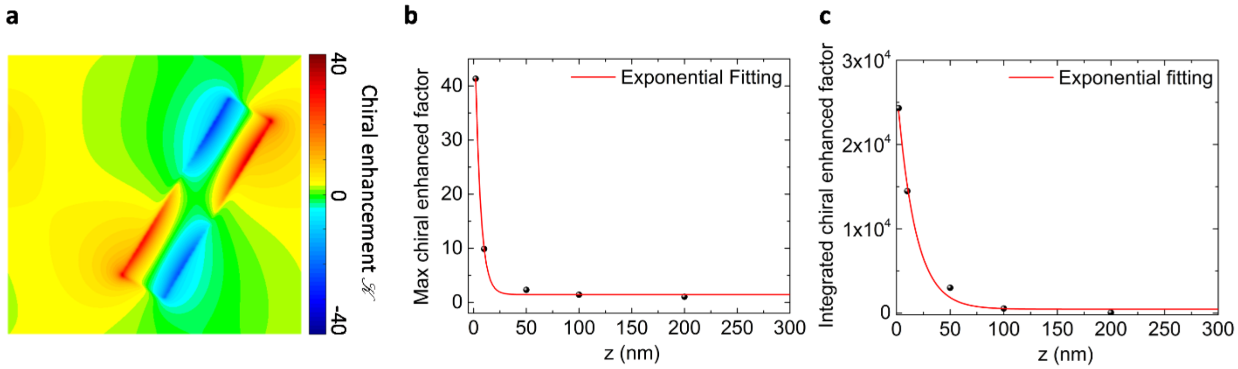

Supplementary Figure 3| Optical chiral enhancement. a, optical chiral enhancement at 2 nm above the top layer of the twisted metamaterials. **b**, maximum of the chiral enhancement factor

decays as a function of distance away from the metamaterials. The red curve is exponential fitting of the simulated data. **c**, Integrated optical chiral enhancement factor with exponential fitting.

Supplementary Note 6. Atomic force microscope (AFM) images for estimating total numbers of molecules in the sensing area

To demonstrate the validity of our estimation of the molecular numbers, we show uniform and monolayer coverage of molecules after spin-coating. We performed AFM scans on a silicon surface. The reasons for choosing a silicon surface as a reference are: 1) it has a low surface roughness, and 2) it has a native oxide layer (a silicon dioxide layer with 1~5 nm thickness). 87.8% of our device area is also silicon dioxide. Therefore, we assume that the silicon dioxide area will capture a similar amount of molecules as the silicon wafer under the same spin coating condition. With the information from x-ray crystallography,[7] our measurements together with ellipsometry measurement suggest a monolayer protein coverage on our device. Using image-processing software (ImageJ), we estimate that one unit cell area of the metamaterial is covered by ~44±7 molecules, corresponding to ~55 zeptomoles of molecules within the entire imaging area of 26 μm by 2.6 μm.

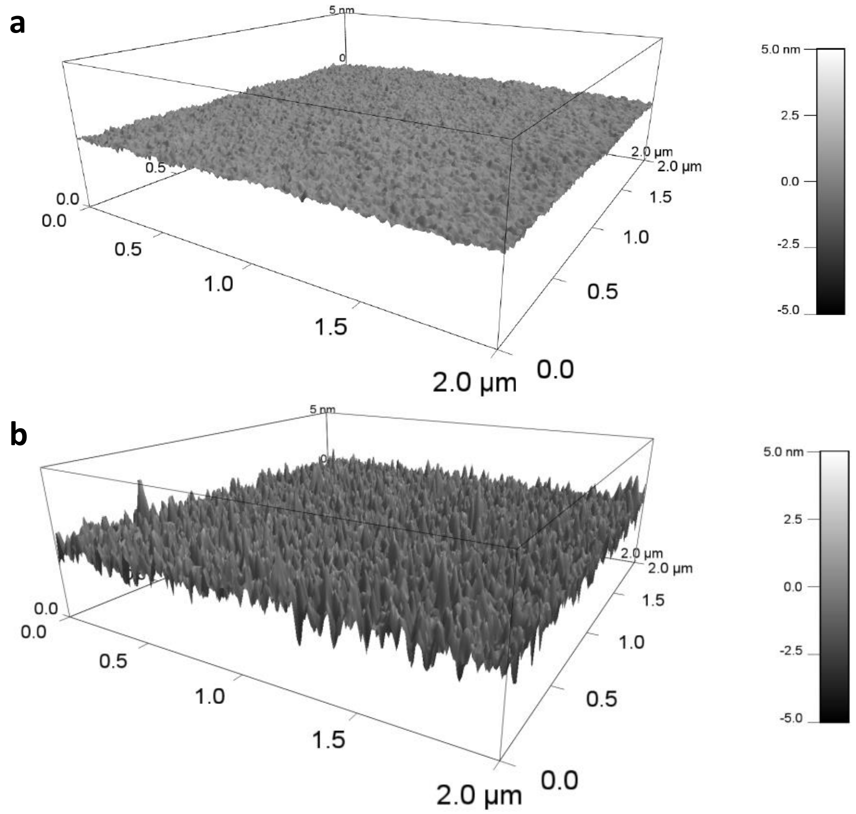

Supplementary Figure 4| Atomic force microscope images of a reference silicon surface. a, before spin-coating ConA molecules. **b**, after spin-coating ConA molecules. The scan area is 2 µm by 2 µm. The scale bar indicates the height of the surface.

Supplementary References

1. I.V. Lindell, A.H. Sihvola, S.A. Tretyakov and A.J. Viitanen, *Electromagnetic Waves in Chiral and Bi-Isotropic Media*, Artech House, London, 1994.
2. Tang, Y. Q. & Cohen, A. E. Optical chirality and its interaction with matter. *Physical Review Letters* **104**, 163901, (2010).

3. Schaferling, M., Dregely, D., Hentschel, M. & Giessen, H. Tailoring enhanced optical chirality: design principles for chiral plasmonic nanostructures. *Physical Review X* **2**, 031010, (2012).
4. Meinzer, N., Hendry, E. & Barnes, W. L. Probing the chiral nature of electromagnetic fields surrounding plasmonic nanostructures. *Physical Review B* **88**, 041407, (2013).
5. Kay, C. The presence of β-structure in Concanavalin A, *Febs Letters* **9**, 78-80, (1970).
6. Ayama, R. *et al.* Determination of self-association of Irinotecan Hydrochloride (CPT-11) in aqueous solution. *Chemical and Pharmaceutical Bulletin* **40**, 2810, (1992).
7. Hardman, K. D., Wood, M. K., Schiffer, M., Edmundson, A. B. & Ainsworth, C. F. Structure of Concanavalin A at 4.25-Angstrom Resolution. P*roceedings of the National Academy of Sciences of the United States of America* **68**, 1393-1397, (1971).
